# Incidence of End-Stage Renal Disease in the Turkish-Cypriot Population of Northern Cyprus: A Population Based Study

**DOI:** 10.1371/journal.pone.0054394

**Published:** 2013-01-17

**Authors:** Thomas M. F. Connor, D. Deren Oygar, Daniel P. Gale, Retha Steenkamp, Dorothea Nitsch, Guy H. Neild, Patrick H. Maxwell

**Affiliations:** 1 UCL Division of Medicine and Centre for Nephrology, University College London, London, United Kingdom; 2 Nicosia State Hospital, Burhan Nalbantoglu General Hospital, Nicosia, North Cyprus; 3 UK Renal Registry, Southmead Hospital, Bristol, United Kingdom; 4 London School of Hygiene and Tropical Medicine, London, United Kingdom; University of Florida, United States of America

## Abstract

**Background:**

This is the first report of the incidence and causes of end-stage renal disease (ESRD) of the Turkish-Cypriot population in Northern Cyprus.

**Methods:**

Data were collected over eight consecutive years (2004–2011) from all those starting renal replacement therapy (RRT) in this population. Crude and age-standardised incidence at 90 days was calculated and comparisons made with other national registries. We collected DNA from the entire prevalent population. As an initial experiment we looked for two genetic causes of ESRD that have been reported in Greek Cypriots.

**Results:**

Crude and age-standardised incidence at 90 days was 234 and 327 per million population (pmp) per year, respectively. The mean age was 63, and 62% were male. The age-adjusted prevalence of RRT in Turkish-Cypriots was 1543 pmp on 01/01/2011. The incidence of RRT is higher than other countries reporting to the European Renal Association – European Dialysis and Transplant Association, with the exception of Turkey. Diabetes is a major cause of ESRD in those under 65, accounting for 36% of incident cases followed by 30% with uncertain aetiology. 18% of the incident population had a family history of ESRD. We identified two families with thin basement membrane nephropathy caused by a mutation in *COL4A3*, but no new cases of CFHR5 nephropathy.

**Conclusions:**

This study provides the first estimate of RRT incidence in the Turkish-Cypriot population, describes the contribution of different underlying diagnoses to ESRD, and provides a basis for healthcare policy planning.

## Introduction

In recent years end-stage renal disease (ESRD) has become an increasing public health challenge for high and middle income countries, with an associated escalation of the cost of providing renal replacement therapy (RRT) [1]–[4]. The collection of accurate epidemiological data is of great importance for healthcare policy planning [1]–[3]. This is especially true for places where the infrastructure delivering RRT is improving, such as the island of Cyprus.

There are considerable differences in the incidence and prevalence of RRT within Europe. Registry data demonstrate a North-South gradient, with higher incidence of RRT and lower mortality around the Mediterranean [2], [5]. Several factors have been suggested to contribute to this variation [6], [7]. In order to understand the aetiology of ESRD and chronic kidney disease, which affects many more people, it is important to determine the primary renal diagnosis [8]. ESRD attributed to type 2 diabetes and hypertension continues to rise throughout the world, and this is increasingly true for countries such as Cyprus [2], [9]–[11].

Cyprus is an island in the eastern Mediterranean that has been occupied by a series of historical powers, in particular the Greeks and Ottoman Turks. The Cypriot population is genetically distinct from mainland populations of either Greece or Turkey, although environmental factors, such as diet and lifestyle are broadly similar [12], [13].

Turkish-Cypriots form a distinct ethno-linguistic community centered on Turkish administered Northern Cyprus (TRNC). The aim of this study was to describe the incidence and prevalence of RRT in this ethnically-defined Mediterranean population by type of primary renal disease. To put these data into context we compared these data with reported RRT incidence from Greece, Turkey, and the white population of England. An important, and probably unique, aspect of our registry is that every RRT patient has provided a DNA sample for research. Because the Greek and Turkish communities share many genetic characteristics [14], [15], we initially undertook genetic testing for two conditions that have been identified in Greek Cypriots, CFHR5 nephropathy and thin basement membrane nephropathy [16], [17].

## Materials and Methods

### Ethics Statement

This study was approved by the ethics committee of Lefkosa Burhan Nalbantoğlu State Hospital. All participants provided informed consent in writing, in accordance with the Declaration of Helsinki.

### Study Population

The study was conducted at Nicosia State Hospital in Northern Cyprus. Nicosia State Hospital is a multi-specialty tertiary care hospital, and provides renal services to the whole of Turkish administered Northern Cyprus (TRNC). All citizens of TRNC are entitled to free RRT, regardless of ethnicity. All patients with symptomatic chronic kidney disease (CKD) from within this population present either through the village practitioners, private hospitals or directly to Nicosia State Hospital. Details of every individual admitted with acute or chronic renal failure in the last decade were collected with a unique identifier number, thus preventing any duplication of records or redundancy of referrals. ESRD was defined as chronic renal disease with an eGFR of <10 ml/min/1.73 m^2^; but patients with diabetic nephropathy often started RRT with eGFR<15 ml/min/1.73 m^2^.

The most recent census of the TRNC population in 2006 showed that of 178,031 *de jure* citizens, 120,007 were born to parents who were both themselves born in Cyprus, providing an effective measure of the size of the ethnic Turkish-Cypriot population that has been applied throughout this paper [18].

### Variables

Basic demographic data (age at ESRD, sex, ethnic group, and probable diagnosis) were recorded for all patients receiving RRT at day 1, and again after 90 days on the renal replacement programme. Diabetic nephropathy was defined as ESRD in the presence of diabetes without evidence of an alternative diagnosis. Family history of ESRD was defined as a first or second degree relative with ESRD.

### Data Sources for International Comparisons

European data was taken from the European Renal Association – European Dialysis and Transplant Association (ERA-EDTA) Registry report for 2008 [2]. Additional data for Greece, Turkey, and England were taken from the Hellenic Renal Registry (Dr GA Ioannidis personal communication), the National Haemodialysis, Transplantation and Nephrology Registry report of Turkey 2008 [19], and the 2008 Renal Registry Report [1]. The denominator population for international comparisons was calculated from the relevant national statistics [20], [21].

### Statistical Analysis

The incidence of RRT in this population was averaged over eight consecutive calendar years (2004–2011). Age-standardised incidence rates were calculated using the Eurostat EU27 population figures [22]. All comparisons with other populations were made using data for chronic RRT; that is those still on RRT at 90 days. The emphasis of this report will be on age-specific comparisons as these are not affected by age-referral biases. We calculated 95% confidence intervals assuming a Poisson distribution for incidence rates. Statistical analysis was carried out using Stata version 11.

### Genetic Testing of All Adult Patients on RRT in North Cyprus

After informed consent for this study, blood was taken from all patients on renal replacement therapy on 01/01/2011. Genetic analysis was performed at University College London, UK. DNA was extracted from peripheral blood using the QIAamp DNA Blood Mini Kit (Qiagen, Stanford, CA, USA). We designed primers to screen for the G1334E and 3854delG mutation in *COL4A4* and the G871C mutation in *COL4A3* [23]. Screening PCR was also performed to amplify both wild-type and mutant *CFHR5* alleles in a single reaction as described previously [16].

## Results

A total of 225 Turkish-Cypriot patients were maintained on renal replacement therapy beyond 90 days during the study period (01/01/04–31/12/11). More males than females started RRT (139 vs 86), but the age distribution was similar between the sexes (Table 1). 18.2% of the incident population had a first or second degree relative with ESRD, and this rose to 27% in those with ESRD due to uncertain aetiology. The average crude and age-standardised incidence rates at day 1 for this period were 311.4 and 456.9 per million population, respectively. The Turkish-Cypriot case mix at 90 days included 36.0% with ESRD due to diabetic nephropathy, 29.8% with unknown diagnosis, and 3.6% with polycystic kidney disease (Table 2). Tetra primer PCR identified two individuals on RRT with the G871C mutation in COL4A3 previously reported in Greek-Cypriot pedigrees [17]. We did not detect the other previously cited mutations in *COL4A3* and *COL4A4* or the *CFHR5* duplication in the RRT population [16], [17].

**Table 1:** Baseline characteristics of the incident Turkish-Cypriot renal replacement therapy (RRT) population at 90 days.

Characteristic	Category	Number	Percentage
Gender	Male	139	61.7%
	Female	86	38.3%
Median age of adult population at ESRF in years(lower and upper quartile)	Male	63 (54,69)	
	Female	64 (52,74)	
Modality of RRT at presentation	Haemodialysis	205	91.1%
	Peritoneal dialysis	10	4.4%
	Renal transplant	8	3.5%
Co-morbidities	Diabetes	93	41.3%
	Hypertension	145	64.4%
	Family History	41	18.2%
Mutation Analysis	COL4A3 (G871C)	2	0.9%
	COL4A3 (G1334E)	0	0%
	COL4A4 (3856delC)	0	0%
	CFHR5 duplication	0	0%

**Table 2:** Provisional renal diagnosis in the Turkish-Cypriot renal replacement therapy (RRT) population at 90 days, by incidence per million population and percentage, in comparison with 2008 registry data for Greece, Turkey, and the UK.

	Turkish-Cypriots		Greece		Turkey		England whites	
Diagnosis	pmp	%	pmp	%	pmp	%	pmp	%
Diabetes	84.4	36.0%	53.5	30.3%	61.6	29.9%	24.5	19.5%
Glomerulonephritis	29.2*	12.4%*	14.8	8.4%	16.4	7.9%	13.5	10.7%
Hypertension	1.0	0.4%	19.1	10.8%	53.4	25.9%	6.8	5.4%
Renal Vascular Disease	12.5	5.3%	3.0	1.7%	2.8	1.3%	7.8	6.2%
Data not available	0.0	0.0%	0.0	0.0%	3.1	1.5%	9.8	7.8%
Other identified Category	12.5	5.3%	15.7	8.9%	21.0	10.2%	18.2	14.5%
Pyelonephritis	16.7	7.1%	9.9	5.6%	7.6	3.7%	9.7	7.7%
Polycystic Kidney	8.3	3.6%	6.7	3.8%	7.8	3.8%	10.1	8.0%
Uncertain aetiology	69.8	29.8%	53.9	30.5%	32.5	15.7%	25.5	20.3%
Total incidence at day 91	234.4	100.0%	176.7	100.0%	206.2	100.0%	125.9	100.0%

* Includes presumed glomerulonephritis not biopsy proven.

The majority (91.1%) of Turkish-Cypriot patients started RRT on haemodialysis; only a few started peritoneal dialysis (4.4%) or had pre-emptive renal transplantation (3.5%) (Table 1). The crude and age-standardised prevalence of RRT in Turkish-Cypriots was 1216 and 1543 pmp, respectively. The median age of the Turkish-Cypriot prevalent population was 60 for both men and women, and the mean duration on RRT was 5.0 years. The majority of patients on RRT in Northern Cyprus are treated with hemodialysis, which is carried out in two centers, Nicosia and Famagusta. The proportion of prevalent patients on peritoneal dialysis (14%) is similar to other countries [24]. Renal transplantation is performed in either Turkey or the Republic of Cyprus (Greek Cypriot), and benefits a high proportion of patients (prevalence 376 pmp).

**Figure 1:**
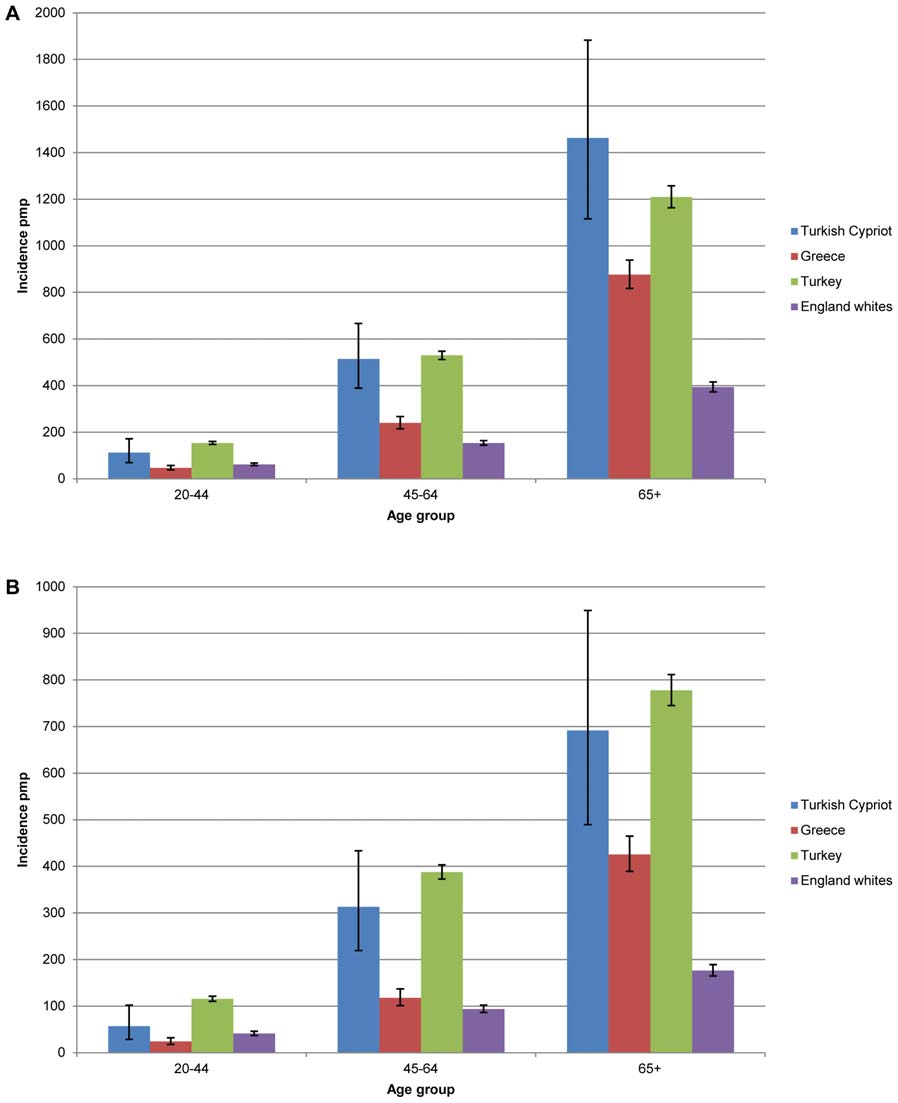
Incidence of renal replacement therapy (RRT) at 90 days by age and gender. Incidence of RRT at 90 days in Turkish Cypriots compared with 2008 registry data for Greece, Turkey, and English whites in males (A), and females (B). Error bars indicate 95% confidence intervals.

We carried out international comparisons. Turkey, Greece, and Tunisia have an age-adjusted incidence rate at day 90 of 349.1, 159.5, and 238.7 pmp respectively [2]. In comparison the crude and age-adjusted incidence rates of chronic RRT for Turkish-Cypriots were 234.4 and 327.2 per million population, respectively. Figure 1 shows that the incidence of RRT in each age group in Turkish-Cypriots is comparable to that seen in Turkey. Despite the small number of cases, the incidence of RRT in the 45–64 age group in Turkish-Cypriots (412.0 pmp) is significantly higher than that seen in the white population of England (123.2 pmp).

There is a high rate of RRT for ESRD attributed to diabetic nephropathy in all eastern Mediterranean countries (84.4 pmp in Turkish-Cypriots, 53.5 pmp in Greece, and 61.6 pmp in Turkey) (Table 2). Table 2 also shows that the code ‘uncertain aetiology’ is more common in the Turkish-Cypriot registry (69.8 pmp) than in other populations. However there were lower levels of hypertensive nephropathy in Turkish-Cypriots than in Greece or Turkey (1.0 pmp vs 19.1 pmp and 53.4 pmp respectively). The incidence of polycystic kidney disease was broadly similar across all registries (range 6.7–10.1 pmp).

The incidence of RRT in the minority Turkish population of Northern Cyprus is broadly in line with the rate seen in Turkish-Cypriots. An additional 76 Turkish patients were maintained on renal replacement therapy beyond 90 days during the study period, giving an overall incidence of 211.3 pmp in the *de jure* population of Northern Cyprus. However data on this population is less accurate due to significant migration to and from the mainland of Turkey [18].

## Discussion

### High Incidence of RRT

This study presents the first population-based RRT incidence figure from Cyprus, and reveals a high incidence of RRT that is RRT is higher than other countries reporting to the ERA-EDTA, with the exception of Turkey [2]. Diabetes is a major cause of ESRD overall and specifically in those under 65, with rates comparable to those seen in the USA [3]. We found that the high incidence of RRT in Turkish-Cypriots is not due to the specific mutations in *COL4A3, COL4A4,* and *CFHR5* assessed in this study [16], [17]. Finally, a third of Turkish-Cypriot patients start RRT with unknown primary diagnosis. This highlights both the need for earlier detection of these cases and the possibility that there may be other uncharacterised conditions causing ESRD in this population.

### Diabetic Nephropathy

**Figure 2:**
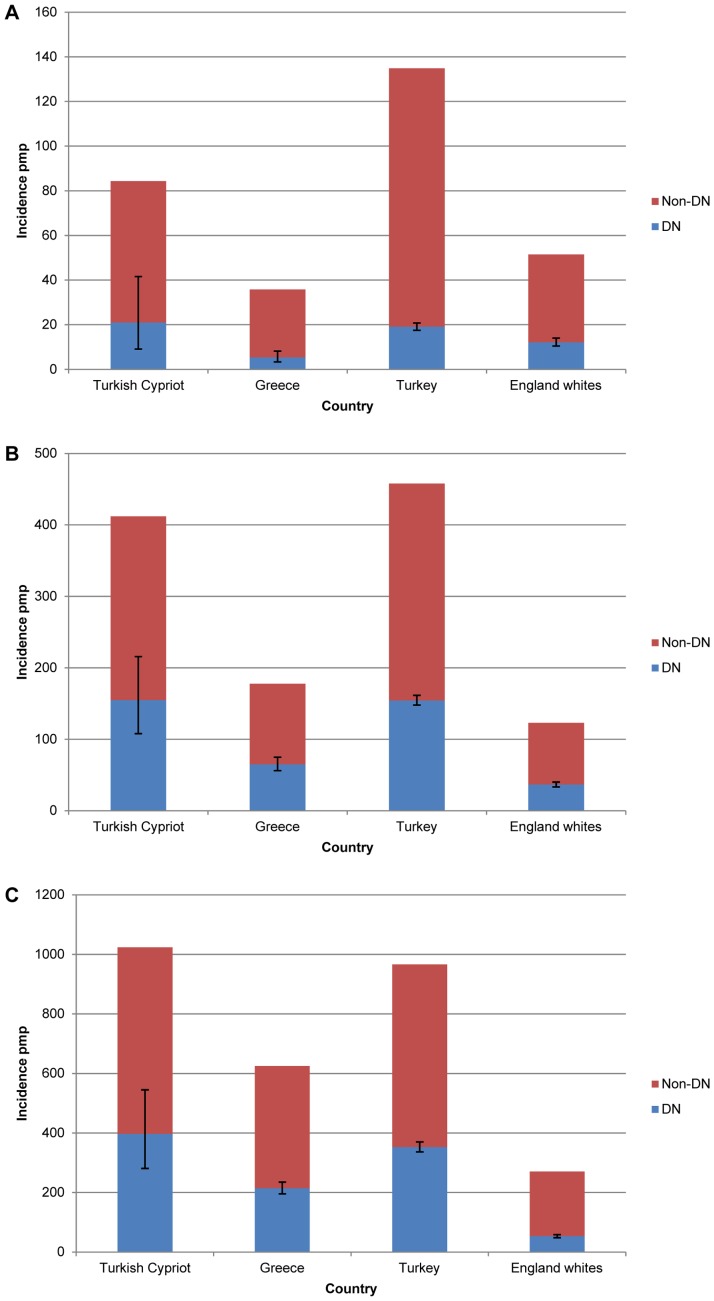
Incidence of renal replacement therapy (RRT) for end stage renal disease (ESRD) due to diabetic nephropathy. Incidence of RRT for ESRD due to diabetic nephropathy (DN) and all other causes (Non-DN) at 90 days in Turkish Cypriots compared with 2008 registry data for Greece, Turkey, and English whites in 20–44 year olds (A), 45–64 year-olds (B), and those over 65 years (C). Error bars indicate 95% confidence intervals.

The Turkish-Cypriot case-mix, with a high incidence of RRT for ESRD due to diabetic nephropathy, is similar to that seen in Turkey and Greece (Table 2). Diabetes is also a common cause of ESRD in developing countries around the Eastern Mediterranean, such as Egypt, Kuwait, Lebanon and Saudi Arabia [11]. The incidence of RRT for ESRD attributed to diabetic nephropathy seen in Turkish-Cypriot less than 65 years old is striking (Figure 2). Currently data concerning the incidence of diabetes in Turkish-Cypriots is lacking. Diabetes is common in Greek-Cypriots and mainland Turkish patients [25]–[27], but not to the extent seen in some other populations with high levels of diabetic nephropathy, such as the Pima Indians of Arizona [28], [29]. Moreover, childhood levels of obesity in Cyprus are much closer to those seen in other European countries than in the US [30], [31]. It is therefore possible that, in common with other registries, some of these cases reflect a co-occurrence of Type 2 diabetes with ESRD due to an alternative aetiology [32].

### Genetic Renal Disease in the Turkish-Cypriot Population

Congenital factors may also be important in the aetiology of ESRD in this population [33]. Previous estimates of the prevalence of family history of ESRD amongst incident dialysis patients, suggest that 7–15% Caucasians have a first or second-degree relative with ESRD [34], [35]. This proportion is highest in young adults, non-Caucasians, and for those where ESRD is caused by diabetes or hypertension [34]. We observed a similar rate of familial ESRD in Turkish-Cypriot patients on RRT, which was even higher (27%) in the group with unknown diagnosis, suggesting the existence of conditions that are not yet characterised in this population. For comparison, the rate of RRT for ESRD due to polycystic kidney disease was similar across all registries.

In order to assess the contribution of inherited renal disease we collected DNA from the entire Turkish-Cypriot population on RRT. Previous work has demonstrated a number of important founder mutations and significant geographic clustering for several monogenetic diseases affecting the Greek-Cypriot population of Cyprus, including mutations of *COL4A3*, *COL4A4, PKD2,* and *MEFV* [17], [36], [37]. Although the *CFHR5* duplication is common in Greek-Cypriots originating from the southern half of the island [38], we did not detect it in our sample of prevalent patients. However, due to the small size of our sample the allelic frequency may still lie within that observed for Greek Cypriots [39]. The familial clustering and high incidence of RRT in Turkish-Cypriots is therefore due to other monogenetic or polygenic diseases in this population.

### Coding ‘Uncertain Aetiology’

There is a significant group in the Turkish-Cypriot RRT population with unknown diagnosis (69.8 pmp). This group partly reflects late presentation and limited diagnostic investigations. Comparative data from the Eastern Mediterranean is difficult to obtain, and information on primary renal disease is less robust [8], [32] but there is evidence for a reciprocal relationship with coding for hypertension in many registries [8], [32], [40]. The incidence of hypertensive nephropathy is conspicuously lower in Turkish-Cypriots than in neighbouring countries. In the absence of clear diagnostic criteria for hypertensive nephropathy, it may be appropriate to combine ‘hypertension’ with ‘uncertain aetiology’ in Table 2, which would imply that there is no established primary renal diagnosis in 40% of patients on RRT in the Greek, Turkish, and Turkish-Cypriot registries.

The majority of patients with an unknown diagnosis present clinically with minimal proteinuria (<1 g protein/day) and asymptomatic disease, consistent with a pathological process primarily affecting the renal tubules [8] and similar to the features of medullary cystic kidney disease type 1. A number of families presenting this way in the Greek Cypriot population have shown linkage to the region 1q21, but the gene responsible has not yet been identified [41]. It is possible that the medullary cystic phenotype reflects the final common pathway of a number of genetic and environmental factors that are common in this population.

### Alternative Explanations for the Incidence of RRT in Turkish-Cypriots

Regional variations in RRT incidence may reflect both genetic and environmental factors [7], [28], [42]. Macroeconomic factors that influence regional variations in RRT incidence include per capita GDP and health-care expenditure [7]. Northern Cyprus has a developing economy, with per-capita GDP that is 76% of that in the Republic of Cyprus, and it is dependent on aid from the Turkish government [43]. RRT has only recently become widely available in TRNC and there is no long-term provision of private dialysis. Economic factors also influence the management of CKD and co-morbidities, as well as the competing risk of mortality, in the general population [7]. The high incidence of RRT in the Turkish-Cypriot population may therefore reflect suboptimal management of diabetes, hypertension, and associated complications. This has significance for healthcare planning on the island, and underlines the importance of prospective assessment of kidney function in this population.

### Wider Relevance of these Findings

Our findings have several implications. First, these data highlight the need to examine the care of diabetics who are at risk of renal disease in the Turkish-Cypriot population, particularly in young people, to prevent rising rates of RRT. To this end, we are examining case records of patients who were diagnosed with diabetic nephropathy and reached ESRD at a young age. Second, there is a large diaspora of Turkish-Cypriots who may carry with them an increased risk of ESRD. Third, this study provides a template for other adult registries across the Middle-East, and highlights the proportion of patients with unknown diagnosis [8]. It is hoped that with greater access to diagnostic investigations this number will be reduced further.

The strength of this study is that we have a complete dataset from the study period, including DNA samples from the entire prevalent RRT population. Many countries with new RRT programs, such as Bangladesh or Malaysia, show increasing take-up of services with time [24]. However the incidence rate, mean age, and sex-ratio of patients on RRT at 90 days shown in Table 1 are broadly in line with other European populations [2]. Moreover, by examining incidence at 90-days in those aged <65 years we have sought to avoid misclassification and bias due to referral patterns, availability of RRT, and prevalence of co-morbidities.

The main limitations of this study are factors affecting the calculation of incidence rates. This study used the same definition of ethnicity as the 2006 census, and the size of the population remained stable over the study period. Referral bias is unlikely as RRT is freely available to all citizens, and because all ERSD is managed through one centre we were able to achieve a remarkably complete dataset.

In conclusion, this study provides a complete dataset of RRT in the Turkish-Cypriot population and shows that incidence and prevalence of RRT are high. Diabetes is a major cause of ESRD overall and specifically in those under 65. With the prevalence of diabetes and hypertension projected to rise further, facilities to target the earlier stages of diabetes and CKD need to be developed further in this population. Earlier identification of CKD together with long-term follow-up will enable more accurate determination of renal diagnosis before the onset of ESRD. Familial renal disease is common in this population, and this study represents the first complete collection of DNA from an ethnically-defined population on RRT. This population therefore provides an opportunity to look for genetic factors associated with an increased risk of ESRD in Cyprus. The high prevalence of RRT in Turkish-Cypriots has implications for healthcare policy planning.
